# Supplementary material for: Modeling glioblastoma heterogeneity as a dynamic network of cell states
Source: Mol Syst Biol. 2021 Sep 16;17(9):e10105. doi: 10.15252/msb.202010105 (PMC8444284; doi:10.15252/msb.202010105)
Supplement: Supplementary file 5 — Source Data for Figure 3 [file MSB-17-e10105-s001.zip › Figure3A_sourcedata/GSEA_3065/nosigdb_state3.GseaPreranked.1622718426028/DOROSZKO_U3180MG_CLUSTER_2.html]

Details for gene set DOROSZKO\_U3180MG\_CLUSTER\_2[GSEA]

|  || Dataset | state3 |
| Phenotype | NoPhenotypeAvailable |
| Upregulated in class | na\_pos |
| GeneSet | DOROSZKO\_U3180MG\_CLUSTER\_2 |
| Enrichment Score (ES) | 0.6968467 |
| Normalized Enrichment Score (NES) | 3.2216582 |
| Nominal p-value | 0.0 |
| FDR q-value | 0.0 |
| FWER p-Value | 0.0 |
Table: GSEA Results Summary

  

Fig 1: Enrichment plot: DOROSZKO\_U3180MG\_CLUSTER\_2      
 Profile of the Running ES Score & Positions of GeneSet Members on the Rank Ordered List

  

| PROBE | GENE SYMBOL | GENE\_TITLE | RANK IN GENE LIST | RANK METRIC SCORE | RUNNING ES | CORE ENRICHMENT || 1 | HSP90B1 |  |  | 7 | 0.563 | 0.0073 | Yes |
| 2 | PDIA6 |  |  | 8 | 0.549 | 0.0152 | Yes |
| 3 | B2M |  |  | 11 | 0.538 | 0.0227 | Yes |
| 4 | PTPRZ1 |  |  | 14 | 0.532 | 0.0302 | Yes |
| 5 | SYNE2 |  |  | 16 | 0.529 | 0.0377 | Yes |
| 6 | APLP2 |  |  | 20 | 0.506 | 0.0446 | Yes |
| 7 | LMAN1 |  |  | 21 | 0.496 | 0.0518 | Yes |
| 8 | CANX |  |  | 27 | 0.479 | 0.0581 | Yes |
| 9 | CALR |  |  | 30 | 0.473 | 0.0647 | Yes |
| 10 | CD9 |  |  | 32 | 0.473 | 0.0714 | Yes |
| 11 | GPM6A |  |  | 37 | 0.460 | 0.0776 | Yes |
| 12 | HSPA5 |  |  | 43 | 0.447 | 0.0834 | Yes |
| 13 | DYNC1H1 |  |  | 47 | 0.437 | 0.0894 | Yes |
| 14 | APP |  |  | 49 | 0.437 | 0.0955 | Yes |
| 15 | NCAM1 |  |  | 52 | 0.430 | 0.1015 | Yes |
| 16 | CCDC47 |  |  | 54 | 0.424 | 0.1075 | Yes |
| 17 | PDIA3 |  |  | 55 | 0.419 | 0.1135 | Yes |
| 18 | SPTBN1 |  |  | 61 | 0.394 | 0.1186 | Yes |
| 19 | ATP6AP2 |  |  | 69 | 0.380 | 0.1233 | Yes |
| 20 | HNRNPU |  |  | 71 | 0.373 | 0.1285 | Yes |
| 21 | DST |  |  | 80 | 0.365 | 0.1328 | Yes |
| 22 | LRPAP1 |  |  | 81 | 0.361 | 0.1380 | Yes |
| 23 | NASP |  |  | 85 | 0.360 | 0.1429 | Yes |
| 24 | FUS |  |  | 87 | 0.358 | 0.1479 | Yes |
| 25 | BSG |  |  | 98 | 0.347 | 0.1517 | Yes |
| 26 | KCNQ1OT1 |  |  | 99 | 0.347 | 0.1567 | Yes |
| 27 | DDOST |  |  | 106 | 0.343 | 0.1610 | Yes |
| 28 | MFGE8 |  |  | 107 | 0.341 | 0.1659 | Yes |
| 29 | RCN2 |  |  | 110 | 0.336 | 0.1705 | Yes |
| 30 | SYT11 |  |  | 111 | 0.335 | 0.1753 | Yes |
| 31 | SON |  |  | 116 | 0.326 | 0.1795 | Yes |
| 32 | PDIA4 |  |  | 120 | 0.324 | 0.1839 | Yes |
| 33 | SEMA5A |  |  | 121 | 0.324 | 0.1885 | Yes |
| 34 | CALD1 |  |  | 124 | 0.322 | 0.1929 | Yes |
| 35 | ACSL3 |  |  | 130 | 0.319 | 0.1969 | Yes |
| 36 | DDX5 |  |  | 134 | 0.316 | 0.2011 | Yes |
| 37 | SLC16A1 |  |  | 138 | 0.314 | 0.2053 | Yes |
| 38 | SNTG1 |  |  | 140 | 0.314 | 0.2097 | Yes |
| 39 | PPIB |  |  | 142 | 0.313 | 0.2141 | Yes |
| 40 | PCMT1 |  |  | 148 | 0.310 | 0.2180 | Yes |
| 41 | ITGB1 |  |  | 150 | 0.310 | 0.2224 | Yes |
| 42 | LRP1 |  |  | 152 | 0.309 | 0.2267 | Yes |
| 43 | LAMP2 |  |  | 154 | 0.309 | 0.2310 | Yes |
| 44 | VMP1 |  |  | 156 | 0.308 | 0.2354 | Yes |
| 45 | MACF1 |  |  | 160 | 0.305 | 0.2394 | Yes |
| 46 | TMEM165 |  |  | 163 | 0.304 | 0.2435 | Yes |
| 47 | PRCP |  |  | 166 | 0.303 | 0.2477 | Yes |
| 48 | ADGRG1 |  |  | 168 | 0.301 | 0.2519 | Yes |
| 49 | SF3B1 |  |  | 180 | 0.295 | 0.2548 | Yes |
| 50 | TM9SF2 |  |  | 189 | 0.291 | 0.2581 | Yes |
| 51 | DDX17 |  |  | 191 | 0.290 | 0.2621 | Yes |
| 52 | SEC61A1 |  |  | 193 | 0.290 | 0.2662 | Yes |
| 53 | SAR1B |  |  | 195 | 0.287 | 0.2702 | Yes |
| 54 | PRKCSH |  |  | 197 | 0.286 | 0.2742 | Yes |
| 55 | GOLGB1 |  |  | 198 | 0.286 | 0.2784 | Yes |
| 56 | GLG1 |  |  | 201 | 0.283 | 0.2822 | Yes |
| 57 | NKTR |  |  | 202 | 0.282 | 0.2863 | Yes |
| 58 | LAPTM4A |  |  | 208 | 0.278 | 0.2897 | Yes |
| 59 | RDH11 |  |  | 212 | 0.277 | 0.2933 | Yes |
| 60 | SELENOS |  |  | 213 | 0.276 | 0.2973 | Yes |
| 61 | LARS |  |  | 214 | 0.276 | 0.3013 | Yes |
| 62 | SLC3A2 |  |  | 215 | 0.276 | 0.3053 | Yes |
| 63 | SFPQ |  |  | 219 | 0.275 | 0.3089 | Yes |
| 64 | RRBP1 |  |  | 220 | 0.274 | 0.3128 | Yes |
| 65 | KTN1 |  |  | 225 | 0.272 | 0.3163 | Yes |
| 66 | ADAM9 |  |  | 229 | 0.272 | 0.3198 | Yes |
| 67 | RCN1 |  |  | 233 | 0.270 | 0.3234 | Yes |
| 68 | POR |  |  | 239 | 0.269 | 0.3267 | Yes |
| 69 | RSRP1 |  |  | 247 | 0.265 | 0.3296 | Yes |
| 70 | DDRGK1 |  |  | 248 | 0.264 | 0.3335 | Yes |
| 71 | TNC |  |  | 249 | 0.264 | 0.3373 | Yes |
| 72 | COL6A1 |  |  | 254 | 0.261 | 0.3406 | Yes |
| 73 | PNN |  |  | 256 | 0.260 | 0.3442 | Yes |
| 74 | MEG3 |  |  | 258 | 0.260 | 0.3478 | Yes |
| 75 | NOVA1 |  |  | 270 | 0.253 | 0.3502 | Yes |
| 76 | SMCHD1 |  |  | 275 | 0.252 | 0.3533 | Yes |
| 77 | DDX18 |  |  | 279 | 0.250 | 0.3566 | Yes |
| 78 | ARGLU1 |  |  | 280 | 0.250 | 0.3602 | Yes |
| 79 | CDH2 |  |  | 283 | 0.249 | 0.3635 | Yes |
| 80 | LAMP1 |  |  | 287 | 0.248 | 0.3667 | Yes |
| 81 | CD164 |  |  | 288 | 0.247 | 0.3703 | Yes |
| 82 | LUC7L3 |  |  | 289 | 0.247 | 0.3739 | Yes |
| 83 | VCAN |  |  | 293 | 0.245 | 0.3771 | Yes |
| 84 | MATN2 |  |  | 318 | 0.239 | 0.3776 | Yes |
| 85 | ATP6V0B |  |  | 319 | 0.238 | 0.3811 | Yes |
| 86 | MFSD10 |  |  | 321 | 0.238 | 0.3844 | Yes |
| 87 | ITGAV |  |  | 322 | 0.238 | 0.3878 | Yes |
| 88 | PCM1 |  |  | 325 | 0.237 | 0.3910 | Yes |
| 89 | SF1 |  |  | 327 | 0.235 | 0.3942 | Yes |
| 90 | PRKCA |  |  | 328 | 0.234 | 0.3976 | Yes |
| 91 | ASPH |  |  | 332 | 0.234 | 0.4006 | Yes |
| 92 | CCDC14 |  |  | 334 | 0.234 | 0.4039 | Yes |
| 93 | MPZL1 |  |  | 340 | 0.231 | 0.4066 | Yes |
| 94 | N4BP2L2 |  |  | 343 | 0.231 | 0.4097 | Yes |
| 95 | ZNF638 |  |  | 346 | 0.230 | 0.4128 | Yes |
| 96 | GON4L |  |  | 349 | 0.230 | 0.4159 | Yes |
| 97 | MYO6 |  |  | 363 | 0.227 | 0.4176 | Yes |
| 98 | AKAP9 |  |  | 368 | 0.226 | 0.4204 | Yes |
| 99 | GUSB |  |  | 369 | 0.226 | 0.4237 | Yes |
| 100 | CTNNB1 |  |  | 373 | 0.224 | 0.4265 | Yes |
| 101 | SPTAN1 |  |  | 376 | 0.223 | 0.4295 | Yes |
| 102 | TNFRSF12A |  |  | 380 | 0.223 | 0.4324 | Yes |
| 103 | SNRNP200 |  |  | 385 | 0.222 | 0.4351 | Yes |
| 104 | HM13 |  |  | 386 | 0.222 | 0.4383 | Yes |
| 105 | RIF1 |  |  | 391 | 0.220 | 0.4410 | Yes |
| 106 | AIG1 |  |  | 393 | 0.219 | 0.4440 | Yes |
| 107 | CTSD |  |  | 398 | 0.219 | 0.4467 | Yes |
| 108 | ZEB2 |  |  | 401 | 0.218 | 0.4496 | Yes |
| 109 | P4HTM |  |  | 402 | 0.218 | 0.4527 | Yes |
| 110 | KANSL1 |  |  | 412 | 0.216 | 0.4548 | Yes |
| 111 | FGFR1 |  |  | 419 | 0.215 | 0.4571 | Yes |
| 112 | WSB1 |  |  | 435 | 0.212 | 0.4584 | Yes |
| 113 | CHD1 |  |  | 436 | 0.212 | 0.4614 | Yes |
| 114 | DDR1 |  |  | 454 | 0.208 | 0.4624 | Yes |
| 115 | RBM25 |  |  | 460 | 0.207 | 0.4648 | Yes |
| 116 | ITGB8 |  |  | 463 | 0.206 | 0.4675 | Yes |
| 117 | PJA2 |  |  | 465 | 0.205 | 0.4704 | Yes |
| 118 | MAGI1 |  |  | 476 | 0.202 | 0.4721 | Yes |
| 119 | FKBP10 |  |  | 481 | 0.201 | 0.4745 | Yes |
| 120 | TMEM30A |  |  | 484 | 0.201 | 0.4771 | Yes |
| 121 | ATP6AP1 |  |  | 487 | 0.201 | 0.4798 | Yes |
| 122 | KMT2A |  |  | 491 | 0.200 | 0.4823 | Yes |
| 123 | CREB5 |  |  | 497 | 0.199 | 0.4846 | Yes |
| 124 | SLC25A37 |  |  | 500 | 0.199 | 0.4872 | Yes |
| 125 | PHF14 |  |  | 504 | 0.198 | 0.4897 | Yes |
| 126 | REV3L |  |  | 506 | 0.198 | 0.4925 | Yes |
| 127 | OS9 |  |  | 510 | 0.197 | 0.4949 | Yes |
| 128 | M6PR |  |  | 520 | 0.196 | 0.4967 | Yes |
| 129 | TTC14 |  |  | 522 | 0.196 | 0.4994 | Yes |
| 130 | SPTLC1 |  |  | 523 | 0.196 | 0.5022 | Yes |
| 131 | CCNL1 |  |  | 529 | 0.194 | 0.5044 | Yes |
| 132 | SERINC1 |  |  | 530 | 0.194 | 0.5072 | Yes |
| 133 | EPM2AIP1 |  |  | 531 | 0.194 | 0.5100 | Yes |
| 134 | DPP6 |  |  | 534 | 0.194 | 0.5126 | Yes |
| 135 | ASH1L |  |  | 535 | 0.194 | 0.5154 | Yes |
| 136 | SDC3 |  |  | 539 | 0.194 | 0.5178 | Yes |
| 137 | GRN |  |  | 551 | 0.192 | 0.5193 | Yes |
| 138 | RGS12 |  |  | 560 | 0.191 | 0.5211 | Yes |
| 139 | ZRANB2 |  |  | 564 | 0.191 | 0.5235 | Yes |
| 140 | GPATCH8 |  |  | 566 | 0.190 | 0.5261 | Yes |
| 141 | THOC2 |  |  | 573 | 0.189 | 0.5281 | Yes |
| 142 | SREK1 |  |  | 578 | 0.189 | 0.5303 | Yes |
| 143 | EFHC1 |  |  | 581 | 0.189 | 0.5328 | Yes |
| 144 | SERPINH1 |  |  | 588 | 0.188 | 0.5348 | Yes |
| 145 | GPBP1 |  |  | 594 | 0.187 | 0.5369 | Yes |
| 146 | PTX3 |  |  | 605 | 0.185 | 0.5384 | Yes |
| 147 | HECTD1 |  |  | 607 | 0.185 | 0.5409 | Yes |
| 148 | CLSTN1 |  |  | 614 | 0.184 | 0.5429 | Yes |
| 149 | NPDC1 |  |  | 617 | 0.184 | 0.5453 | Yes |
| 150 | CHD9 |  |  | 620 | 0.184 | 0.5477 | Yes |
| 151 | TUFM |  |  | 626 | 0.183 | 0.5497 | Yes |
| 152 | ATRX |  |  | 638 | 0.180 | 0.5510 | Yes |
| 153 | LIMA1 |  |  | 639 | 0.180 | 0.5536 | Yes |
| 154 | KMT2C |  |  | 646 | 0.179 | 0.5555 | Yes |
| 155 | TMEM87A |  |  | 656 | 0.177 | 0.5569 | Yes |
| 156 | SPAG9 |  |  | 667 | 0.176 | 0.5583 | Yes |
| 157 | CHD6 |  |  | 670 | 0.176 | 0.5606 | Yes |
| 158 | RPAP2 |  |  | 673 | 0.175 | 0.5629 | Yes |
| 159 | TAF1D |  |  | 675 | 0.175 | 0.5653 | Yes |
| 160 | SLC35F1 |  |  | 711 | 0.168 | 0.5635 | Yes |
| 161 | OFD1 |  |  | 714 | 0.168 | 0.5657 | Yes |
| 162 | PNISR |  |  | 718 | 0.168 | 0.5677 | Yes |
| 163 | PRPF4B |  |  | 724 | 0.167 | 0.5695 | Yes |
| 164 | SUMF2 |  |  | 727 | 0.167 | 0.5717 | Yes |
| 165 | SPG7 |  |  | 729 | 0.167 | 0.5740 | Yes |
| 166 | GPRC5B |  |  | 730 | 0.166 | 0.5764 | Yes |
| 167 | IFNGR1 |  |  | 733 | 0.166 | 0.5786 | Yes |
| 168 | CTSL |  |  | 735 | 0.166 | 0.5808 | Yes |
| 169 | SLC1A3 |  |  | 741 | 0.165 | 0.5826 | Yes |
| 170 | PRRC2B |  |  | 744 | 0.165 | 0.5848 | Yes |
| 171 | CAND1 |  |  | 750 | 0.164 | 0.5865 | Yes |
| 172 | ATF6B |  |  | 763 | 0.162 | 0.5874 | Yes |
| 173 | KIF1B |  |  | 767 | 0.161 | 0.5894 | Yes |
| 174 | SLC39A1 |  |  | 771 | 0.161 | 0.5913 | Yes |
| 175 | FNIP1 |  |  | 780 | 0.160 | 0.5927 | Yes |
| 176 | PLPP3 |  |  | 786 | 0.159 | 0.5944 | Yes |
| 177 | FSTL1 |  |  | 790 | 0.159 | 0.5963 | Yes |
| 178 | ATP2A2 |  |  | 792 | 0.158 | 0.5985 | Yes |
| 179 | HNRNPH1 |  |  | 793 | 0.158 | 0.6007 | Yes |
| 180 | PODXL2 |  |  | 806 | 0.157 | 0.6016 | Yes |
| 181 | RAB6A |  |  | 809 | 0.157 | 0.6036 | Yes |
| 182 | HSPA4 |  |  | 824 | 0.155 | 0.6041 | Yes |
| 183 | NOP56 |  |  | 829 | 0.155 | 0.6059 | Yes |
| 184 | JMJD1C |  |  | 838 | 0.154 | 0.6071 | Yes |
| 185 | PTK2 |  |  | 841 | 0.153 | 0.6091 | Yes |
| 186 | COPA |  |  | 853 | 0.152 | 0.6100 | Yes |
| 187 | NEAT1 |  |  | 855 | 0.152 | 0.6121 | Yes |
| 188 | CCDC66 |  |  | 861 | 0.151 | 0.6136 | Yes |
| 189 | NOC2L |  |  | 864 | 0.151 | 0.6156 | Yes |
| 190 | RTN4 |  |  | 869 | 0.151 | 0.6173 | Yes |
| 191 | SPPL2A |  |  | 870 | 0.151 | 0.6195 | Yes |
| 192 | CYR61 |  |  | 873 | 0.150 | 0.6214 | Yes |
| 193 | FAM133B |  |  | 881 | 0.150 | 0.6227 | Yes |
| 194 | GOLIM4 |  |  | 892 | 0.149 | 0.6236 | Yes |
| 195 | ZNF207 |  |  | 896 | 0.149 | 0.6254 | Yes |
| 196 | ACAP3 |  |  | 900 | 0.149 | 0.6272 | Yes |
| 197 | ZNF302 |  |  | 910 | 0.148 | 0.6283 | Yes |
| 198 | ESF1 |  |  | 921 | 0.146 | 0.6292 | Yes |
| 199 | NIN |  |  | 933 | 0.145 | 0.6299 | Yes |
| 200 | HSPH1 |  |  | 937 | 0.144 | 0.6317 | Yes |
| 201 | PTCD3 |  |  | 947 | 0.143 | 0.6326 | Yes |
| 202 | SLC12A9 |  |  | 957 | 0.141 | 0.6336 | Yes |
| 203 | HMGXB4 |  |  | 962 | 0.141 | 0.6351 | Yes |
| 204 | STAT3 |  |  | 965 | 0.141 | 0.6369 | Yes |
| 205 | WDR73 |  |  | 981 | 0.140 | 0.6371 | Yes |
| 206 | CLU |  |  | 988 | 0.139 | 0.6384 | Yes |
| 207 | GALR1 |  |  | 989 | 0.139 | 0.6404 | Yes |
| 208 | ADAR |  |  | 991 | 0.139 | 0.6423 | Yes |
| 209 | RNMT |  |  | 995 | 0.138 | 0.6439 | Yes |
| 210 | DLL3 |  |  | 999 | 0.138 | 0.6456 | Yes |
| 211 | RBM28 |  |  | 1010 | 0.138 | 0.6464 | Yes |
| 212 | JAG1 |  |  | 1011 | 0.138 | 0.6484 | Yes |
| 213 | MPHOSPH8 |  |  | 1030 | 0.136 | 0.6481 | Yes |
| 214 | XBP1 |  |  | 1036 | 0.135 | 0.6495 | Yes |
| 215 | RBM39 |  |  | 1043 | 0.135 | 0.6507 | Yes |
| 216 | SPECC1 |  |  | 1048 | 0.134 | 0.6522 | Yes |
| 217 | ZNF655 |  |  | 1051 | 0.134 | 0.6539 | Yes |
| 218 | FJX1 |  |  | 1071 | 0.133 | 0.6535 | Yes |
| 219 | CNOT4 |  |  | 1082 | 0.132 | 0.6542 | Yes |
| 220 | MYCBP2 |  |  | 1084 | 0.132 | 0.6560 | Yes |
| 221 | NSD1 |  |  | 1085 | 0.132 | 0.6579 | Yes |
| 222 | AUP1 |  |  | 1093 | 0.130 | 0.6589 | Yes |
| 223 | CTSF |  |  | 1103 | 0.129 | 0.6597 | Yes |
| 224 | UNC50 |  |  | 1105 | 0.129 | 0.6614 | Yes |
| 225 | DDX6 |  |  | 1108 | 0.129 | 0.6631 | Yes |
| 226 | CCDC82 |  |  | 1137 | 0.127 | 0.6615 | Yes |
| 227 | CDC42BPA |  |  | 1141 | 0.127 | 0.6630 | Yes |
| 228 | EXOC7 |  |  | 1142 | 0.127 | 0.6648 | Yes |
| 229 | SNRNP70 |  |  | 1148 | 0.126 | 0.6660 | Yes |
| 230 | SRSF5 |  |  | 1158 | 0.125 | 0.6667 | Yes |
| 231 | SETD5 |  |  | 1164 | 0.125 | 0.6679 | Yes |
| 232 | UBR5 |  |  | 1173 | 0.124 | 0.6688 | Yes |
| 233 | DAAM1 |  |  | 1211 | 0.121 | 0.6660 | Yes |
| 234 | RNF115 |  |  | 1226 | 0.120 | 0.6661 | Yes |
| 235 | MTF2 |  |  | 1230 | 0.119 | 0.6674 | Yes |
| 236 | ZNF397 |  |  | 1231 | 0.119 | 0.6692 | Yes |
| 237 | B4GAT1 |  |  | 1238 | 0.119 | 0.6701 | Yes |
| 238 | SECISBP2 |  |  | 1243 | 0.118 | 0.6714 | Yes |
| 239 | GPATCH2 |  |  | 1244 | 0.118 | 0.6731 | Yes |
| 240 | ZNF800 |  |  | 1248 | 0.118 | 0.6744 | Yes |
| 241 | CCDC88A |  |  | 1249 | 0.118 | 0.6761 | Yes |
| 242 | CSNK2A1 |  |  | 1252 | 0.118 | 0.6776 | Yes |
| 243 | HEPN1 |  |  | 1255 | 0.118 | 0.6790 | Yes |
| 244 | ZNF292 |  |  | 1262 | 0.117 | 0.6800 | Yes |
| 245 | PABPN1 |  |  | 1273 | 0.117 | 0.6805 | Yes |
| 246 | DDX46 |  |  | 1276 | 0.116 | 0.6819 | Yes |
| 247 | PUS7L |  |  | 1288 | 0.116 | 0.6823 | Yes |
| 248 | DHX36 |  |  | 1320 | 0.114 | 0.6802 | Yes |
| 249 | ARHGEF2 |  |  | 1323 | 0.114 | 0.6816 | Yes |
| 250 | TNRC6B |  |  | 1326 | 0.114 | 0.6830 | Yes |
| 251 | SRSF7 |  |  | 1328 | 0.113 | 0.6845 | Yes |
| 252 | SRSF10 |  |  | 1345 | 0.112 | 0.6842 | Yes |
| 253 | PRRC2A |  |  | 1346 | 0.112 | 0.6858 | Yes |
| 254 | EWSR1 |  |  | 1354 | 0.111 | 0.6865 | Yes |
| 255 | SACS |  |  | 1360 | 0.111 | 0.6875 | Yes |
| 256 | ANKRD12 |  |  | 1368 | 0.110 | 0.6883 | Yes |
| 257 | EXOC4 |  |  | 1372 | 0.110 | 0.6895 | Yes |
| 258 | TMUB2 |  |  | 1376 | 0.110 | 0.6907 | Yes |
| 259 | MAT2A |  |  | 1386 | 0.109 | 0.6912 | Yes |
| 260 | TAOK1 |  |  | 1407 | 0.108 | 0.6904 | Yes |
| 261 | NAA15 |  |  | 1413 | 0.107 | 0.6913 | Yes |
| 262 | SLC22A17 |  |  | 1419 | 0.107 | 0.6923 | Yes |
| 263 | PBRM1 |  |  | 1424 | 0.107 | 0.6933 | Yes |
| 264 | ZNF37A |  |  | 1425 | 0.107 | 0.6949 | Yes |
| 265 | SMARCC2 |  |  | 1433 | 0.107 | 0.6956 | Yes |
| 266 | RBM26 |  |  | 1436 | 0.106 | 0.6968 | Yes |
| 267 | GTF2H2 |  |  | 1458 | 0.105 | 0.6958 | No |
| 268 | COLGALT2 |  |  | 1492 | 0.103 | 0.6933 | No |
| 269 | HES1 |  |  | 1501 | 0.103 | 0.6939 | No |
| 270 | USP33 |  |  | 1537 | 0.101 | 0.6911 | No |
| 271 | BDP1 |  |  | 1543 | 0.101 | 0.6919 | No |
| 272 | MED6 |  |  | 1547 | 0.101 | 0.6930 | No |
| 273 | ARHGAP21 |  |  | 1602 | 0.097 | 0.6879 | No |
| 274 | ZBTB20 |  |  | 1632 | 0.095 | 0.6858 | No |
| 275 | EIF2S3 |  |  | 1638 | 0.095 | 0.6866 | No |
| 276 | MSI2 |  |  | 1640 | 0.095 | 0.6878 | No |
| 277 | QTRT1 |  |  | 1661 | 0.093 | 0.6868 | No |
| 278 | USP34 |  |  | 1667 | 0.093 | 0.6875 | No |
| 279 | MUM1 |  |  | 1672 | 0.093 | 0.6884 | No |
| 280 | SRSF2 |  |  | 1697 | 0.092 | 0.6868 | No |
| 281 | LONP2 |  |  | 1707 | 0.091 | 0.6870 | No |
| 282 | TTC37 |  |  | 1721 | 0.090 | 0.6868 | No |
| 283 | DDX3X |  |  | 1750 | 0.089 | 0.6847 | No |
| 284 | ZNF131 |  |  | 1802 | 0.087 | 0.6798 | No |
| 285 | RIC3 |  |  | 1804 | 0.087 | 0.6809 | No |
| 286 | TERF1 |  |  | 1830 | 0.085 | 0.6791 | No |
| 287 | RNF170 |  |  | 1832 | 0.085 | 0.6802 | No |
| 288 | CDK5RAP3 |  |  | 1835 | 0.085 | 0.6812 | No |
| 289 | CTSA |  |  | 1837 | 0.084 | 0.6823 | No |
| 290 | NCOR1 |  |  | 1844 | 0.084 | 0.6828 | No |
| 291 | U2SURP |  |  | 1848 | 0.084 | 0.6836 | No |
| 292 | TRA2A |  |  | 1849 | 0.084 | 0.6848 | No |
| 293 | TRMT13 |  |  | 1852 | 0.084 | 0.6858 | No |
| 294 | KIF21A |  |  | 1855 | 0.084 | 0.6868 | No |
| 295 | MKLN1 |  |  | 1902 | 0.081 | 0.6824 | No |
| 296 | ARID4A |  |  | 1907 | 0.081 | 0.6831 | No |
| 297 | HOOK3 |  |  | 1934 | 0.079 | 0.6811 | No |
| 298 | RSBN1L |  |  | 1940 | 0.079 | 0.6816 | No |
| 299 | GCC2 |  |  | 1944 | 0.078 | 0.6824 | No |
| 300 | TBL1XR1 |  |  | 1949 | 0.078 | 0.6830 | No |
| 301 | PKN2 |  |  | 1956 | 0.078 | 0.6834 | No |
| 302 | PSAP |  |  | 1988 | 0.076 | 0.6808 | No |
| 303 | NCK1 |  |  | 1998 | 0.076 | 0.6808 | No |
| 304 | ELMSAN1 |  |  | 2004 | 0.076 | 0.6813 | No |
| 305 | LUC7L |  |  | 2015 | 0.075 | 0.6812 | No |
| 306 | SRSF6 |  |  | 2027 | 0.075 | 0.6809 | No |
| 307 | TRIM44 |  |  | 2043 | 0.074 | 0.6802 | No |
| 308 | RAB3IP |  |  | 2061 | 0.073 | 0.6792 | No |
| 309 | TMEM59L |  |  | 2066 | 0.073 | 0.6798 | No |
| 310 | ANAPC7 |  |  | 2071 | 0.073 | 0.6804 | No |
| 311 | WDR60 |  |  | 2103 | 0.072 | 0.6776 | No |
| 312 | RBM4B |  |  | 2111 | 0.071 | 0.6778 | No |
| 313 | IGFBP3 |  |  | 2112 | 0.071 | 0.6789 | No |
| 314 | EPN2 |  |  | 2164 | 0.070 | 0.6737 | No |
| 315 | RIMKLB |  |  | 2185 | 0.069 | 0.6723 | No |
| 316 | DDB1 |  |  | 2190 | 0.069 | 0.6728 | No |
| 317 | ABHD12 |  |  | 2241 | 0.066 | 0.6677 | No |
| 318 | TCF4 |  |  | 2253 | 0.066 | 0.6674 | No |
| 319 | CTTN |  |  | 2280 | 0.065 | 0.6652 | No |
| 320 | SAFB2 |  |  | 2289 | 0.065 | 0.6651 | No |
| 321 | HERPUD1 |  |  | 2299 | 0.064 | 0.6650 | No |
| 322 | MYC |  |  | 2324 | 0.064 | 0.6630 | No |
| 323 | MARK3 |  |  | 2328 | 0.063 | 0.6636 | No |
| 324 | PRPF38B |  |  | 2336 | 0.063 | 0.6636 | No |
| 325 | CWC25 |  |  | 2338 | 0.063 | 0.6644 | No |
| 326 | PTBP1 |  |  | 2359 | 0.062 | 0.6629 | No |
| 327 | NELFCD |  |  | 2426 | 0.060 | 0.6558 | No |
| 328 | LRP10 |  |  | 2436 | 0.059 | 0.6556 | No |
| 329 | IFRD1 |  |  | 2444 | 0.059 | 0.6556 | No |
| 330 | SRSF1 |  |  | 2450 | 0.059 | 0.6558 | No |
| 331 | NAV1 |  |  | 2451 | 0.059 | 0.6567 | No |
| 332 | TTC19 |  |  | 2499 | 0.057 | 0.6518 | No |
| 333 | FAM53C |  |  | 2503 | 0.057 | 0.6523 | No |
| 334 | MEIS2 |  |  | 2506 | 0.057 | 0.6529 | No |
| 335 | AFF4 |  |  | 2549 | 0.055 | 0.6486 | No |
| 336 | ARFGAP2 |  |  | 2564 | 0.055 | 0.6477 | No |
| 337 | BICD1 |  |  | 2566 | 0.055 | 0.6484 | No |
| 338 | BCLAF1 |  |  | 2603 | 0.053 | 0.6448 | No |
| 339 | GATAD1 |  |  | 2654 | 0.051 | 0.6395 | No |
| 340 | NSUN5 |  |  | 2659 | 0.051 | 0.6398 | No |
| 341 | WEE1 |  |  | 2687 | 0.050 | 0.6372 | No |
| 342 | HSPD1 |  |  | 2700 | 0.050 | 0.6365 | No |
| 343 | B3GAT3 |  |  | 2705 | 0.050 | 0.6367 | No |
| 344 | GPAA1 |  |  | 2719 | 0.050 | 0.6359 | No |
| 345 | DPF2 |  |  | 2726 | 0.049 | 0.6359 | No |
| 346 | BAZ2B |  |  | 2803 | 0.047 | 0.6274 | No |
| 347 | HEPACAM |  |  | 2847 | 0.046 | 0.6228 | No |
| 348 | ARHGEF7 |  |  | 2867 | 0.045 | 0.6212 | No |
| 349 | KNOP1 |  |  | 2880 | 0.045 | 0.6204 | No |
| 350 | PHF20 |  |  | 2968 | 0.042 | 0.6105 | No |
| 351 | TLE4 |  |  | 2975 | 0.042 | 0.6104 | No |
| 352 | ABI2 |  |  | 2977 | 0.041 | 0.6109 | No |
| 353 | DNM1L |  |  | 2983 | 0.041 | 0.6109 | No |
| 354 | NPAS3 |  |  | 2988 | 0.041 | 0.6110 | No |
| 355 | IST1 |  |  | 3002 | 0.041 | 0.6100 | No |
| 356 | STX16 |  |  | 3016 | 0.041 | 0.6090 | No |
| 357 | CCS |  |  | 3063 | 0.040 | 0.6040 | No |
| 358 | DHX30 |  |  | 3064 | 0.039 | 0.6046 | No |
| 359 | DPF3 |  |  | 3123 | 0.038 | 0.5982 | No |
| 360 | TMEM120A |  |  | 3153 | 0.037 | 0.5952 | No |
| 361 | PRPSAP1 |  |  | 3163 | 0.037 | 0.5946 | No |
| 362 | CYHR1 |  |  | 3176 | 0.036 | 0.5937 | No |
| 363 | GDAP1 |  |  | 3303 | 0.033 | 0.5790 | No |
| 364 | TLK1 |  |  | 3326 | 0.032 | 0.5768 | No |
| 365 | NEMF |  |  | 3360 | 0.032 | 0.5733 | No |
| 366 | TCF25 |  |  | 3431 | 0.030 | 0.5653 | No |
| 367 | HSPA1B |  |  | 3440 | 0.029 | 0.5647 | No |
| 368 | TAOK3 |  |  | 3466 | 0.029 | 0.5621 | No |
| 369 | COMMD2 |  |  | 3499 | 0.028 | 0.5587 | No |
| 370 | TIMM44 |  |  | 3501 | 0.028 | 0.5590 | No |
| 371 | CLK1 |  |  | 3511 | 0.028 | 0.5583 | No |
| 372 | ANAPC5 |  |  | 3522 | 0.028 | 0.5575 | No |
| 373 | CDC37 |  |  | 3535 | 0.027 | 0.5564 | No |
| 374 | SLC25A36 |  |  | 3552 | 0.027 | 0.5549 | No |
| 375 | DDX42 |  |  | 3554 | 0.027 | 0.5551 | No |
| 376 | SUPT20H |  |  | 3596 | 0.026 | 0.5506 | No |
| 377 | ACADVL |  |  | 3600 | 0.026 | 0.5506 | No |
| 378 | MLF1 |  |  | 3601 | 0.026 | 0.5509 | No |
| 379 | ABHD5 |  |  | 3610 | 0.025 | 0.5503 | No |
| 380 | ABCE1 |  |  | 3645 | 0.024 | 0.5466 | No |
| 381 | PBXIP1 |  |  | 3663 | 0.024 | 0.5449 | No |
| 382 | PSRC1 |  |  | 3675 | 0.024 | 0.5439 | No |
| 383 | CEBPZ |  |  | 3687 | 0.023 | 0.5429 | No |
| 384 | CPEB4 |  |  | 3702 | 0.023 | 0.5415 | No |
| 385 | ARHGAP5 |  |  | 3722 | 0.022 | 0.5396 | No |
| 386 | ORAI2 |  |  | 3757 | 0.021 | 0.5358 | No |
| 387 | MCL1 |  |  | 3848 | 0.019 | 0.5252 | No |
| 388 | ZNF680 |  |  | 3850 | 0.019 | 0.5253 | No |
| 389 | TUBGCP2 |  |  | 3882 | 0.018 | 0.5218 | No |
| 390 | TCERG1 |  |  | 3985 | 0.016 | 0.5098 | No |
| 391 | SRSF11 |  |  | 4003 | 0.015 | 0.5079 | No |
| 392 | DCLK2 |  |  | 4033 | 0.014 | 0.5046 | No |
| 393 | RPRD1A |  |  | 4046 | 0.014 | 0.5034 | No |
| 394 | GNL3 |  |  | 4103 | 0.012 | 0.4968 | No |
| 395 | DTNA |  |  | 4203 | 0.009 | 0.4850 | No |
| 396 | FOXN2 |  |  | 4204 | 0.009 | 0.4851 | No |
| 397 | RB1CC1 |  |  | 4216 | 0.009 | 0.4839 | No |
| 398 | CHMP4A |  |  | 4249 | 0.008 | 0.4802 | No |
| 399 | GTF2I |  |  | 4264 | 0.008 | 0.4786 | No |
| 400 | KMT2E |  |  | 4295 | 0.007 | 0.4751 | No |
| 401 | SAT2 |  |  | 4311 | 0.006 | 0.4734 | No |
| 402 | DNAJC2 |  |  | 4461 | 0.002 | 0.4554 | No |
| 403 | KMT5B |  |  | 4671 | -0.003 | 0.4303 | No |
| 404 | RFX4 |  |  | 4672 | -0.003 | 0.4303 | No |
| 405 | CELF1 |  |  | 4691 | -0.003 | 0.4282 | No |
| 406 | HAGHL |  |  | 4737 | -0.004 | 0.4228 | No |
| 407 | NCSTN |  |  | 4795 | -0.006 | 0.4160 | No |
| 408 | USP3 |  |  | 4815 | -0.006 | 0.4138 | No |
| 409 | LFNG |  |  | 4861 | -0.007 | 0.4085 | No |
| 410 | JPX |  |  | 4864 | -0.008 | 0.4084 | No |
| 411 | GNPAT |  |  | 4907 | -0.009 | 0.4034 | No |
| 412 | FOXO3 |  |  | 4960 | -0.010 | 0.3973 | No |
| 413 | CRTC3 |  |  | 5065 | -0.013 | 0.3850 | No |
| 414 | SFXN5 |  |  | 5066 | -0.013 | 0.3851 | No |
| 415 | DMAP1 |  |  | 5137 | -0.014 | 0.3769 | No |
| 416 | PSMC6 |  |  | 5139 | -0.014 | 0.3770 | No |
| 417 | AHCYL1 |  |  | 5145 | -0.014 | 0.3766 | No |
| 418 | SPARCL1 |  |  | 5152 | -0.014 | 0.3761 | No |
| 419 | THAP5 |  |  | 5166 | -0.015 | 0.3747 | No |
| 420 | FERMT1 |  |  | 5185 | -0.015 | 0.3728 | No |
| 421 | TIA1 |  |  | 5218 | -0.016 | 0.3691 | No |
| 422 | RAF1 |  |  | 5375 | -0.020 | 0.3506 | No |
| 423 | DTX3 |  |  | 5390 | -0.021 | 0.3492 | No |
| 424 | RBM23 |  |  | 5407 | -0.021 | 0.3476 | No |
| 425 | ST5 |  |  | 5408 | -0.021 | 0.3479 | No |
| 426 | ATXN7L3B |  |  | 5423 | -0.022 | 0.3465 | No |
| 427 | FAM172A |  |  | 5609 | -0.028 | 0.3246 | No |
| 428 | SPSB3 |  |  | 5640 | -0.028 | 0.3214 | No |
| 429 | USP16 |  |  | 5641 | -0.028 | 0.3218 | No |
| 430 | TDG |  |  | 5692 | -0.030 | 0.3162 | No |
| 431 | IP6K2 |  |  | 5699 | -0.030 | 0.3159 | No |
| 432 | CNOT2 |  |  | 5714 | -0.030 | 0.3147 | No |
| 433 | PPP1R16A |  |  | 5721 | -0.030 | 0.3144 | No |
| 434 | NTM |  |  | 5738 | -0.031 | 0.3129 | No |
| 435 | CNTN1 |  |  | 5924 | -0.037 | 0.2911 | No |
| 436 | FASTK |  |  | 6013 | -0.040 | 0.2811 | No |
| 437 | TRA2B |  |  | 6483 | -0.056 | 0.2253 | No |
| 438 | CFLAR |  |  | 6582 | -0.060 | 0.2143 | No |
| 439 | ANK2 |  |  | 6640 | -0.063 | 0.2084 | No |
| 440 | VPS37B |  |  | 6650 | -0.064 | 0.2082 | No |
| 441 | MTA1 |  |  | 6668 | -0.064 | 0.2071 | No |
| 442 | CEBPZOS |  |  | 6699 | -0.066 | 0.2044 | No |
| 443 | PGP |  |  | 6718 | -0.067 | 0.2032 | No |
| 444 | OSBPL1A |  |  | 6733 | -0.068 | 0.2025 | No |
| 445 | YME1L1 |  |  | 6862 | -0.074 | 0.1881 | No |
| 446 | DDIT3 |  |  | 6918 | -0.077 | 0.1826 | No |
| 447 | DNPEP |  |  | 6925 | -0.077 | 0.1830 | No |
| 448 | ARIH2 |  |  | 6948 | -0.078 | 0.1814 | No |
| 449 | IDS |  |  | 6974 | -0.079 | 0.1796 | No |
| 450 | CAMK2D |  |  | 6988 | -0.080 | 0.1791 | No |
| 451 | MTRF1L |  |  | 7074 | -0.085 | 0.1701 | No |
| 452 | IFRD2 |  |  | 7190 | -0.093 | 0.1576 | No |
| 453 | SNHG9 |  |  | 7260 | -0.097 | 0.1507 | No |
| 454 | IRS2 |  |  | 7285 | -0.099 | 0.1492 | No |
| 455 | ARRDC3 |  |  | 7386 | -0.107 | 0.1387 | No |
| 456 | LMNA |  |  | 7412 | -0.109 | 0.1372 | No |
| 457 | IAH1 |  |  | 7529 | -0.120 | 0.1250 | No |
| 458 | FAM120A |  |  | 7624 | -0.130 | 0.1155 | No |
| 459 | EMD |  |  | 7890 | -0.164 | 0.0859 | No |
| 460 | PPP1CB |  |  | 8232 | -0.235 | 0.0482 | No |
| 461 | GADD45A |  |  | 8306 | -0.260 | 0.0431 | No |
| 462 | SAT1 |  |  | 8533 | -0.354 | 0.0210 | No |
| 463 | YBX3 |  |  | 8545 | -0.362 | 0.0249 | No |
Table: GSEA details [plain text format]

  

Fig 2: DOROSZKO\_U3180MG\_CLUSTER\_2: Random ES distribution      
 Gene set null distribution of ES for **DOROSZKO\_U3180MG\_CLUSTER\_2**
